# Occupational differences in mortality and life expectancy persist after retirement and throughout life

**DOI:** 10.1177/14034948221081628

**Published:** 2022-03-24

**Authors:** Ebeling Marcus, Ahlbom Anders, Gustavsson Per, Modig Karin

**Affiliations:** 1 Unit of Epidemiology, Karolinska Institutet, Sweden; 2 Laboratory of Population Health, Max Planck Institute for Demographic Research, Germany; 3 Centre for Occupational and Environmental Medicine, Region Stockholm, Sweden; 4 Unit of Occupational Medicine, Karolinska Institutet, Sweden

**Keywords:** Life expectancy, occupations, old age, mortality differences, lifespan, birth cohorts, partial life expectancy, age-specific death rates

## Abstract

**Aims:**

There are substantial differences in remaining life expectancy at higher ages between occupational groups. These differences may be the effect of work-related exposures, lifestyle factors of workers in specific occupations, socioeconomic position or a combination of this. The scope of this paper is the extent to which occupational differences in remaining life expectancy persist after retirement, which would suggest that occupational exposures alone are not likely to explain all the difference.

**Methods:**

All individuals born between 1925 and 1939 who reported occupational information in the Census 1985 and were residents in Sweden to the end of 2020 or who died were included and followed for death until 2020. The Nordic Classification of Occupations was used to create nine occupational groups. Partial life expectancy and age-specific death rates were applied to examine mortality differentials.

**Results:**

This study showed substantial differences in partial life expectancy across the occupational cohorts with the biggest difference being about 2 years. The mortality differences persisted with increasing age, both when measured as absolute numbers as well as relative numbers.

**Conclusions:**

**The lack of convergence in mortality at high ages suggests that factors associated with lifestyle may play a larger role than occupational factors for the mortality differences between occupational groups at high ages. However, it cannot be ruled out that long-lasting effects of earlier occupational exposures also contribute. Regardless of the exact mechanism, we conclude that there is room for further reduction in mortality at high ages and, thus, for further improvement in life expectancy.**

## Introduction

There are considerable differences in mortality across population groups within any country, often expressed as differences in life expectancy. Such differences relate to education, income, employment, and occupation, as well as other indicators of socioeconomic position. Not only does employment in itself promote health [1], but health inequalities between different occupations are substantial [2]. Hence, life expectancy varies across occupational groups, with those in more affluent jobs being better off [3–5]. A large number of job-related exposures have been shown to increase the risk of disease and of death, including chemicals, asbestos, dusts, physical factors, and psychosocial factors [6–9]. However, occupation is closely linked to socioeconomic position, which has a clear impact on health and mortality [10]. Moreover, occupation is linked to lifestyle factors such as smoking and diet, which are also known to affect health and mortality [11]. For example, in Sweden professional drivers, manufacturing workers and cleaners have a threefold increased risk of type 2 diabetes compared with university teachers and physiotherapists [12]. Thus, mortality differences within the workforce are likely to be a combined effect of job-related exposures, socioeconomic position, and lifestyle factors, that are often impossible to disentangle.

Occupational exposures may cease to affect health when the exposure is discontinued and consequently lead to declining differences in mortality between occupational groups. Alternatively, the effect of occupational exposure may persist into ages beyond retirement age if the exposures accumulate with a long induction period. As for socioeconomic position and lifestyle factors, we know that such factors accumulate over the life course. However, it is important to note that also other dynamics, such as selective mortality (e.g. most frail individuals die off first) irrespective of occupation, could cause a mortality convergence between occupational groups.

Most studies of socioeconomic or occupational differences in health and mortality are performed in working ages. Nevertheless, several studies have also explored socioeconomic differences in old age [13, 14], and in general have found that such differences exist in old age even if they are less pronounced than in younger ages [15–17]. However, studies exploring how differences evolve over age, in a cohort manner, and especially during the transition into retirement are few.

The scope of this paper is the extent to which occupational differences in life expectancy change with age and in particular decrease with increasing age. A special focus is on ages in which retirement is common. There are two lines of thought behind this. First, to the extent that differences in the risk of death are due to ongoing job exposure they can be expected to decrease or vanish after retirement. Second, mortality differences that persist with increasing age and past exit from work may be due to occupational effects on health that do not level off, or they may be due to persisting socioeconomic or lifestyle factors that accumulate over the life course and continue to influence the risk of death, perhaps to the end of life.

## Materials and methods

Data for the study were derived from the Swedish population registers and are described in detail elsewhere [18, 19]. All individuals born between 1925 and 1939 who reported occupational information in the Census 1985 and were residents in the country to the end of 2020 or to death were included. The one-year birth cohorts were lumped into three cohorts to increase sizes. The three cohorts are the birth cohorts 1925–1929, 1930–1934 and 1935–1939. The cohorts were followed up with respect to mortality to 2020. Thus, those born between 1925 and 1929 could be observed between 61 and 91 years of age, those born between 1930 and 1934 between 56 and 86 years, and those born between 1925 and 1926 between 51 and 81 years, see visualisation in Supplemental Figure 1.

The job titles from the Census 1985 were used to distinguish different occupational groups. The job titles were coded according to the Nordic classification of occupations – Swedish standard [20]. This coding scheme distinguishes occupations by hierarchical five-digit codes. We used primarily first-digit codes, although for certain jobs we used two digits to create more sensible groups of reasonable size. Table I presents the resulting occupational groups that the analyses are based on. For men, we distinguished a total of nine occupational groups, while we considered only eight different groups for women because the group of women in ‘civilian protection service work’ was not large enough. For both women and men, the same applies to the groups of ‘military work’ and ‘mining, quarrying, and petroleum work’, which have also been omitted from the analyses. Moreover, we also excluded the individuals with unidentifiable or inadequately reported occupations. Several other options for the occupational groupings were considered, some of which are elaborated in the discussion section.

**Table I.** Grouping of occupations for this study, based on the Nordic classification of occupations – Swedish standard (NYK).

NYK code	Occupation
0	Professional, technical and related work
1	Health and nursing work, social work
2	Administrative, managerial and clerical work
3	Sales work
4	Agricultural, forestry, and fishing work
6	Transport and communications work
7, 8	Production work
90 (only men)	Civilian protective service work
91, 92, 93, 94	Lodging and catering service work Private household work Caretaking and cleaning work Hygiene and personal care work

The group of women in ‘civilian protection service work’ (NYK code 90) have been omitted due to population size. The same applies to the men and women in ‘mining, quarrying, and petroleum extraction work’ (NYK code 5) and ‘military work’ (NYK code 98). We excluded individuals with unidentifiable or inadequately reported occupations (NYK code 99).

Partial life expectancy (PLE) was employed to show differences in mortality across the selected occupational groups. This measure is like standard life expectancy and is calculated by using a similar methodology [21]. PLE is used here because data did not allow follow-up of the cohorts until they were extinct, but only to 2020. PLE is the average number of years an individual of a cohort lives within a specific age range. For instance, those born between 1925 and 1929 are observed between the ages of 61 and 91 years. In the hypothetical case that nobody dies during this age range, everyone would live for 31 years, and the PLE would also be 31 years. However, as individuals die, the PLE will be lower and depend on the magnitude of mortality. The PLE summarises mortality over an age range into one single value, and it implicitly standardises for age, and thus, allows comparisons between different populations. PLEs were calculated for each occupational group, for men and women separately, and for each of the three cohorts.

The question explored in this study is whether the mortality differences between occupations decrease with increasing age or not. To address this question the starting age for the PLE calculations were gradually moved upwards to a higher age. Decreasing occupational mortality differences with increasing starting age of the PLE calculation indicates that mortality in the various occupations converges when cohort members get older. However, moving the starting age to higher ages, and thus, also shrinking the age range will ultimately lead to lower PLEs for all occupations. Therefore, we analysed only standardised differences. The standardisation was performed by dividing the difference between the observed occupation-specific PLE and the minimum PLE across occupations by the difference between the minimum and maximum value of PLE across occupations. We performed the standardisation separately for each age range and sex. This approach was inspired by a procedure used in the life expectancy deprivation index that is part of the human development index [22].

In addition to the PLEs, age and sex-specific death rates were calculated for the various cohorts and occupations based on the number of deaths and number of person-years in each stratum. To reduce fluctuations, the death rates were smoothed using a P-Spline approach in a Poisson generalised additive model before calculating PLE. Smoothing was performed using the mgcv package in R [23]. The data underlying this study are personal data which cannot be shared in detail without ethical permission. Nevertheless, codes and data in aggregated form are openly accessible in https://osf.io/sk4f9/.

## Results

The initial size of the population and exclusions for various reasons are shown in Supplemental Table I. The final size of the three cohorts used for the analyses were 314,414 for the oldest, 347,810 for the middle, and 390,775 for the youngest. Supplemental Table I (men) and Supplemental Table II (women) display the number of deaths and person-years that are the basis for the death rate calculations.

Detailed information about the PLEs at start are available in Supplemental Figure 2. The figure has a panel for each combination of birth cohort and gender. The magnitude of the difference depends on the age of the cohort and on the occupation. There are substantial differences in PLE across the occupational cohorts, with the biggest difference being about 2 years. For the youngest cohort (aged 51–81 years) the highest number is 28.6 years, for women in ‘agricultural, forestry and fishing work’. The lowest number is 27.3 years for ‘production work’. That is a 1.3-year difference in the average number of years lived in this age range. For men in the same cohort the highest number is for ‘health, nursing, and social work’ and is 27.5 years, while the lowest PLE is for ‘catering, caretaking, and care work, 25.5 years, a 2-year difference between the highest and lowest with respect to the average number of years lived in the 31-year-wide age interval.

**Figure 1.**
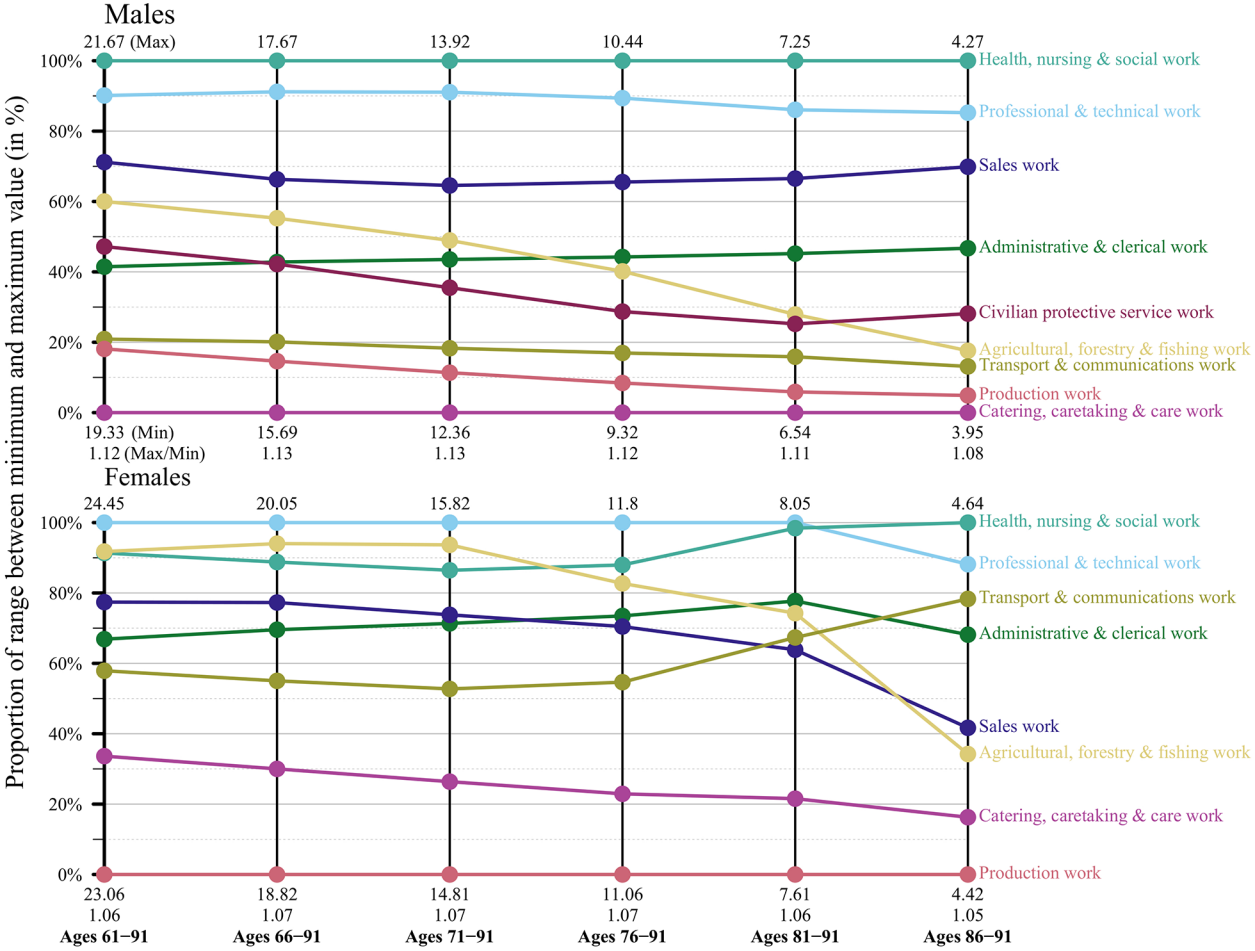
Partial life expectancy differences relative to respective minimum and maximum values by age range and occupational groups, birth cohorts 1925–1929, men and women, Sweden.

**Figure 2.**
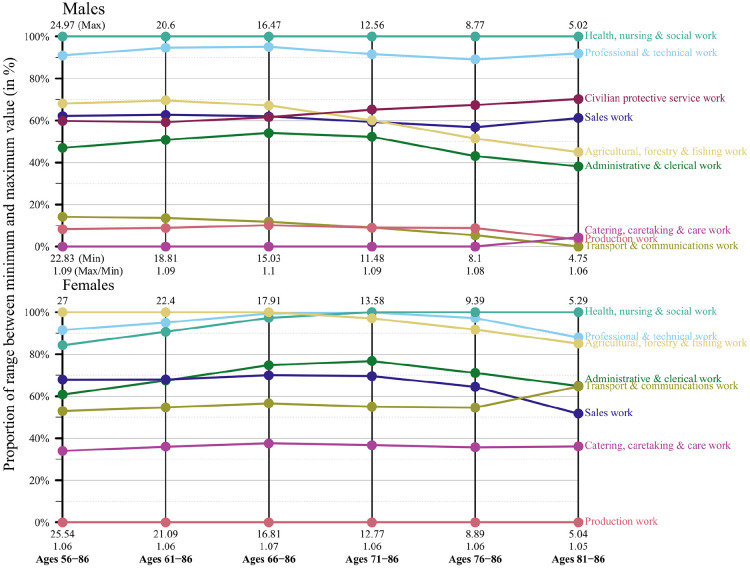
Partial life expectancy differences relative to respective minimum and maximum values by age range and occupational groups, birth cohorts 1930–1934, men and women, Sweden.

**Figure 3.**
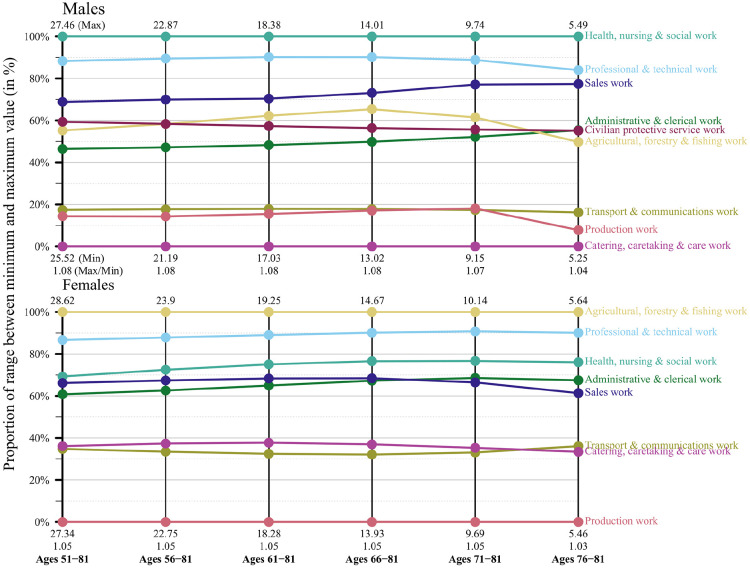
Partial life expectancy differences relative to respective minimum and maximum values by age range and occupational groups, birth cohorts 1935–1939, men and women, Sweden.

Figures 1–3 show for each of the three birth cohorts what happens to the PLE differences between the occupations when the age span is shrinking and compressed towards the oldest end. As described previously, the PLE differences are standardised in this analysis because the maximum value of PLE decreases in the process of moving the calculations to higher ages. The figures display the proportion of the range between the minimum and maximum value that each occupation holds. A horizontal line indicates that an occupation maintains its position relative to the other occupations. Non-horizontal lines would show that the differences in PLE between occupations had changed with increasing age. However, the lines are almost parallel, at least for the two latest birth cohorts. The implication is that the occupations maintain their positions relative to each other.

Figures 1–3 also give the actual values of the PLE for the highest and lowest occupations for every 5 years. For women in the youngest cohort, for example, the occupation with the highest PLE goes down from 28.6 to 5.6 when the calculations are restricted to the highest 5-year age category. For the occupation with the lowest PLE the corresponding change is from 27.3 to 5.5. The ratio between the highest and lowest PLE at the beginning is 28.6/27.3 = 1.05 and at the end 5.6/5.5 = 1.03. For men in this cohort the change in the top occupation is from 27.5 to 5.5 and in the bottom occupation from 25.5 to 5.3. The ratio between top and bottom is therefore 1.08 at the beginning and 1.04 at the end. All these ratios are shown in the figures.

Hence, in absolute numbers the differences in PLE between occupations decrease substantially when restriction is made to the oldest ages, but this follows naturally because the age interval has been compressed and mortality increased because of higher age. However, when measured in relative numbers the occupational differences in PLE that exist at the start of follow-up essentially remain when calculations are restricted to the oldest age.

**Figure 4.**
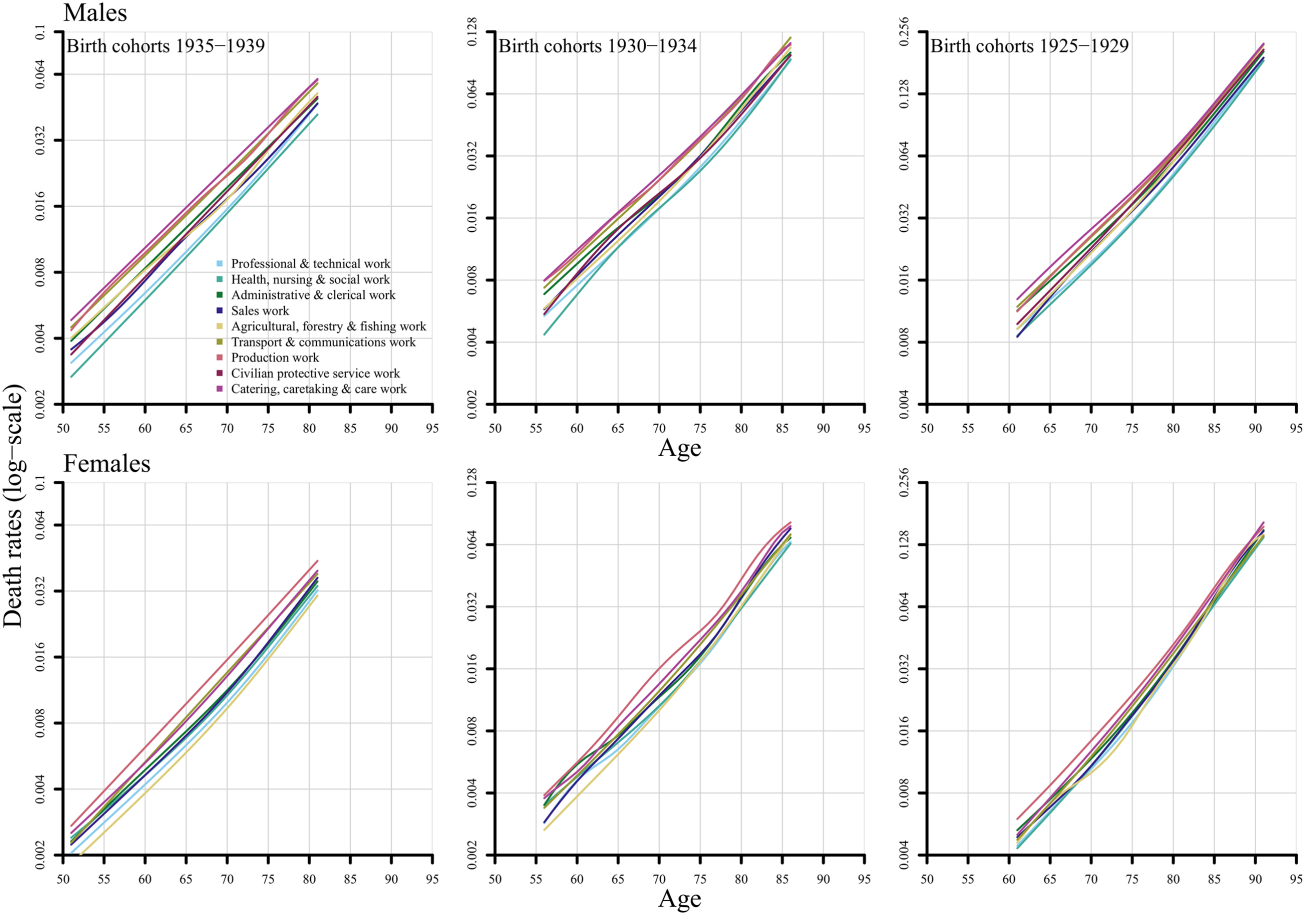
Smoothed age-specific death rates by birth cohort and occupational group, men and women, Sweden.

Figure 4 displays the age and sex-specific death rates for each birth cohort and occupation. These graphs are based on the same data as the PLE figures but present the results differently. The results are the same as those obtained from the PLE figures above, namely that there are consistent differences in death rates between occupations and they persist across the whole age range mostly at similar distances. Similar figures but for each occupation can be found in Supplemental Figures 3 (men) and 4 (women).

## Discussion

This study shows the anticipated differences in mortality between occupational groups, but also that these differences do not vanish with increasing age. Instead, the mortality differences remained essentially the same across the entire included age span, although the choice of scale for comparison played some role. This study utilised three different metrics to assess the occupational differences: the standardised PLE, the ratio between the highest and lowest occupation, and the age and sex-specific death rates. The three various methods gave similar results.

The explanation of the mortality differences within the workforce is likely to be a combination of work- related exposure and socioeconomic position and lifestyle factors. It is important to point out that our approach as well as the underlying data do not allow us to draw any conclusions about the specific role of occupational exposures, or even the relative contribution of each of these factors. However, if there had been a clear decrease in the mortality differences near the typical time of exit from the workforce it would have indicated that work-related exposure was of particular importance, but this was not the case. Instead, the observed differences that are evident all the way to the end of follow-up, that is to 91 years of age in the oldest cohort, are most likelyto be a consequence of work-related exposure that maintains its effect through life or socioeconomic position and/or lifestyle factors of workers in specific occupations, or to a combination of this. Regardless of the explanation, the results imply that there is room for further improvement of mortality, also at high ages. It is assumed that the mortality gap can be reduced towards the lower levels represented by the groups with the longest PLE. Consequently, there is also room for further improvement of life expectancy. This is relevant because mortality reduction at advanced age has been shown to be a requisite for the further increase in life expectancy [24].

The occupational groups in this study were the one-digit groups based on the NYK codes (in one case the two-digit codes). We considered using groupings that more specifically pinpointed work-related exposure. First, we combined job titles based on the three-digit codes into groups with presumably similar exposure situations. However, it was evident after some attempts that the jobs covered by a given job title included too heterogeneous occupational activities for such a grouping to serve its purpose. Second, we attempted to base the grouping of occupations on a job exposure matrix [25]. However, job exposure matrices are better aimed to investigate association with specific diseases and cause-specific mortality and are not optimal for the present study investigating overall mortality. In the end we were satisfied with using the one-digit job titles as the occupational groups because they capture the combination of occupational exposure and socioeconomic factors and because they showed the anticipated mortality differences at the start.

The occupational information was extracted from the 1985 Census and reflects the situation at that time point, rather than during a longer period of follow-up. Although that is a limitation, changes of job during the follow-up period would not have falsely hidden a decrease in mortality differences. Moreover, to the extent that the contrasts in mortality that show up in these analyses are due to socioeconomic factors independent of work, it would have been of value to trace them further back in life to younger ages. However, the available data did not allow that.

## Conclusions

The study confirms the well-established mortality discrepancies across occupations and concludes that there is essentially no sign that these discrepancies disappear or even shrink around the age of retirement or at more advanced ages. Occupational effects on mortality are likely to be a combination of both short and long-term effects, so the lack of convergence in mortality with age after retirement speaks in favour of long-term effects. Regardless of this, these results imply that that there is room for further improvement of mortality at high ages and a continued increase in life expectancy.

## Supplemental Material

sj-docx-1-sjp-10.1177_14034948221081628 – Supplemental material for Occupational differences in mortality and life expectancy persist after retirement and throughout life Click here for additional data file. Supplemental material, sj-docx-1-sjp-10.1177_14034948221081628 for Occupational differences in mortality and life expectancy persist after retirement and throughout life by Ebeling Marcus, Ahlbom Anders, Gustavsson Per and Modig Karin in Scandinavian Journal of Public Health

sj-jpg-2-sjp-10.1177_14034948221081628 – Supplemental material for Occupational differences in mortality and life expectancy persist after retirement and throughout life Click here for additional data file. Supplemental material, sj-jpg-2-sjp-10.1177_14034948221081628 for Occupational differences in mortality and life expectancy persist after retirement and throughout life by Ebeling Marcus, Ahlbom Anders, Gustavsson Per and Modig Karin in Scandinavian Journal of Public Health

sj-jpg-3-sjp-10.1177_14034948221081628 – Supplemental material for Occupational differences in mortality and life expectancy persist after retirement and throughout life Click here for additional data file. Supplemental material, sj-jpg-3-sjp-10.1177_14034948221081628 for Occupational differences in mortality and life expectancy persist after retirement and throughout life by Ebeling Marcus, Ahlbom Anders, Gustavsson Per and Modig Karin in Scandinavian Journal of Public Health

sj-jpg-4-sjp-10.1177_14034948221081628 – Supplemental material for Occupational differences in mortality and life expectancy persist after retirement and throughout life Click here for additional data file. Supplemental material, sj-jpg-4-sjp-10.1177_14034948221081628 for Occupational differences in mortality and life expectancy persist after retirement and throughout life by Ebeling Marcus, Ahlbom Anders, Gustavsson Per and Modig Karin in Scandinavian Journal of Public Health

sj-jpg-5-sjp-10.1177_14034948221081628 – Supplemental material for Occupational differences in mortality and life expectancy persist after retirement and throughout life Click here for additional data file. Supplemental material, sj-jpg-5-sjp-10.1177_14034948221081628 for Occupational differences in mortality and life expectancy persist after retirement and throughout life by Ebeling Marcus, Ahlbom Anders, Gustavsson Per and Modig Karin in Scandinavian Journal of Public Health
