# Supplementary material for: Occupational differences in mortality and life expectancy persist after retirement and throughout life
Source: Scand J Public Health. 2022 Mar 24;51(6):894–901. doi: 10.1177/14034948221081628 (PMC10350732; doi:10.1177/14034948221081628)
Supplement: sj-docx-1-sjp-10.1177_14034948221081628 – Supplemental material for Occupational differences in mortality and life expectancy persist after retirement and throughout life [file sj-docx-1-sjp-10.1177_14034948221081628.docx]

**Supplementary Table 1: Total number of deaths and person years under risk by occupational groups and birth cohorts, males, Sweden.**

|  | **Cohorts 1925-29, Ages 61-91** | | **Cohorts 1930-34, Ages 56-86** | | **Cohorts 1935-39, Ages 51-81** | |
| --- | --- | --- | --- | --- | --- | --- |
| **Occupation** | **Death** | **Person Years** | **Death** | **Person Years** | **Death** | **Person Years** |
| Professional, technical and related work | 30831.0 | 806200.7 | 25306.0 | 1058897.9 | 20253.0 | 1454092.9 |
| Health and nursing work, social work | 5858.0 | 156434.6 | 4420.0 | 189337.1 | 3238.0 | 248053.7 |
| Administrative, managerial and clerical work | 6757.0 | 160111.5 | 4949.0 | 182221.6 | 3277.0 | 200346.9 |
| Sales work | 11763.0 | 295447.5 | 9843.0 | 377628.4 | 7265.0 | 487736.5 |
| Agricultural, forestry, and fishing work | 13483.0 | 324550.7 | 8932.0 | 340982.1 | 5305.0 | 331995.9 |
| Transport and communications work | 12743.0 | 287990.2 | 10740.0 | 357723.6 | 7839.0 | 422538.6 |
| Production work | 48784.0 | 1094756.0 | 42892.0 | 1424313.3 | 33719.0 | 1792382.7 |
| Civilian protective service work | 2441.0 | 57757.6 | 1889.0 | 72673.3 | 1483.0 | 93605.3 |
| Lodging and catering service work, Privat household work, Caretaking and cleaning work, Hygiene and personal care work | 6838.0 | 149091.8 | 5343.0 | 174495.2 | 3908.0 | 198374.4 |

**Supplementary Table 2: Total number of deaths and person years under risk by occupational groups and birth cohorts, females, Sweden.**

|  | **Cohorts 1925-29, Ages 61-91** | | **Cohorts 1930-34, Ages 56-86** | | **Cohorts 1935-39, Ages 51-81** | |
| --- | --- | --- | --- | --- | --- | --- |
| **Occupation** | **Death** | **Person Years** | **Death** | **Person Years** | **Death** | **Person Years** |
| Professional, technical and related work | 25765.0 | 916439.1 | 22056.0 | 1327409.4 | 17046.0 | 1795259.8 |
| Health and nursing work, social work | 2213.0 | 78140.5 | 1549.0 | 93397.0 | 1159.0 | 113750.8 |
| Administrative, managerial and clerical work | 20782.0 | 702925.2 | 15448.0 | 856583.9 | 11030.0 | 1039623.3 |
| Sales work | 10479.0 | 354819.1 | 7985.0 | 436408.1 | 5039.0 | 475495.6 |
| Agricultural, forestry, and fishing work | 3375.0 | 116223.2 | 2256.0 | 135636.3 | 1168.0 | 130229.2 |
| Transport and communications work | 4085.0 | 137456.1 | 3175.0 | 171119.6 | 2119.0 | 177336.6 |
| Production work | 9693.0 | 296121.7 | 7740.0 | 358468.8 | 5270.0 | 388219.3 |
| Lodging and catering service work, Privat household work, Caretaking and cleaning work, Hygiene and personal care work | 26553.0 | 840780.2 | 19119.0 | 964894.7 | 12804.0 | 1072704.9 |
