# Supplementary figures and images for: Occupational differences in mortality and life expectancy persist after retirement and throughout life
Source: Scand J Public Health. 2022 Mar 24;51(6):894–901. doi: 10.1177/14034948221081628 (PMC10350732; doi:10.1177/14034948221081628)

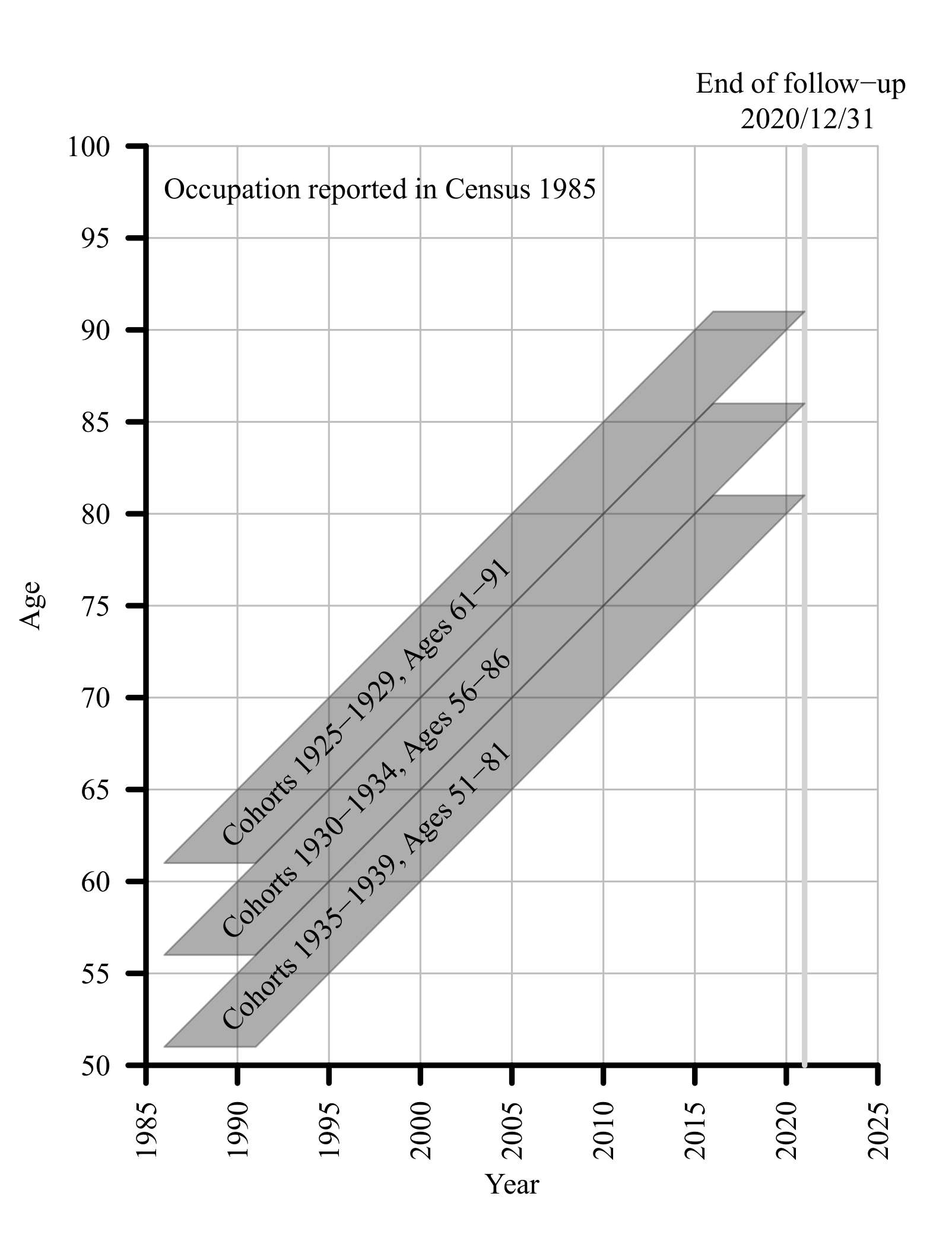

Supplement: sj-jpg-2-sjp-10.1177_14034948221081628 – Supplemental material for Occupational differences in mortality and life expectancy persist after retirement and throughout life [file sj-jpg-2-sjp-10.1177_14034948221081628.jpg]

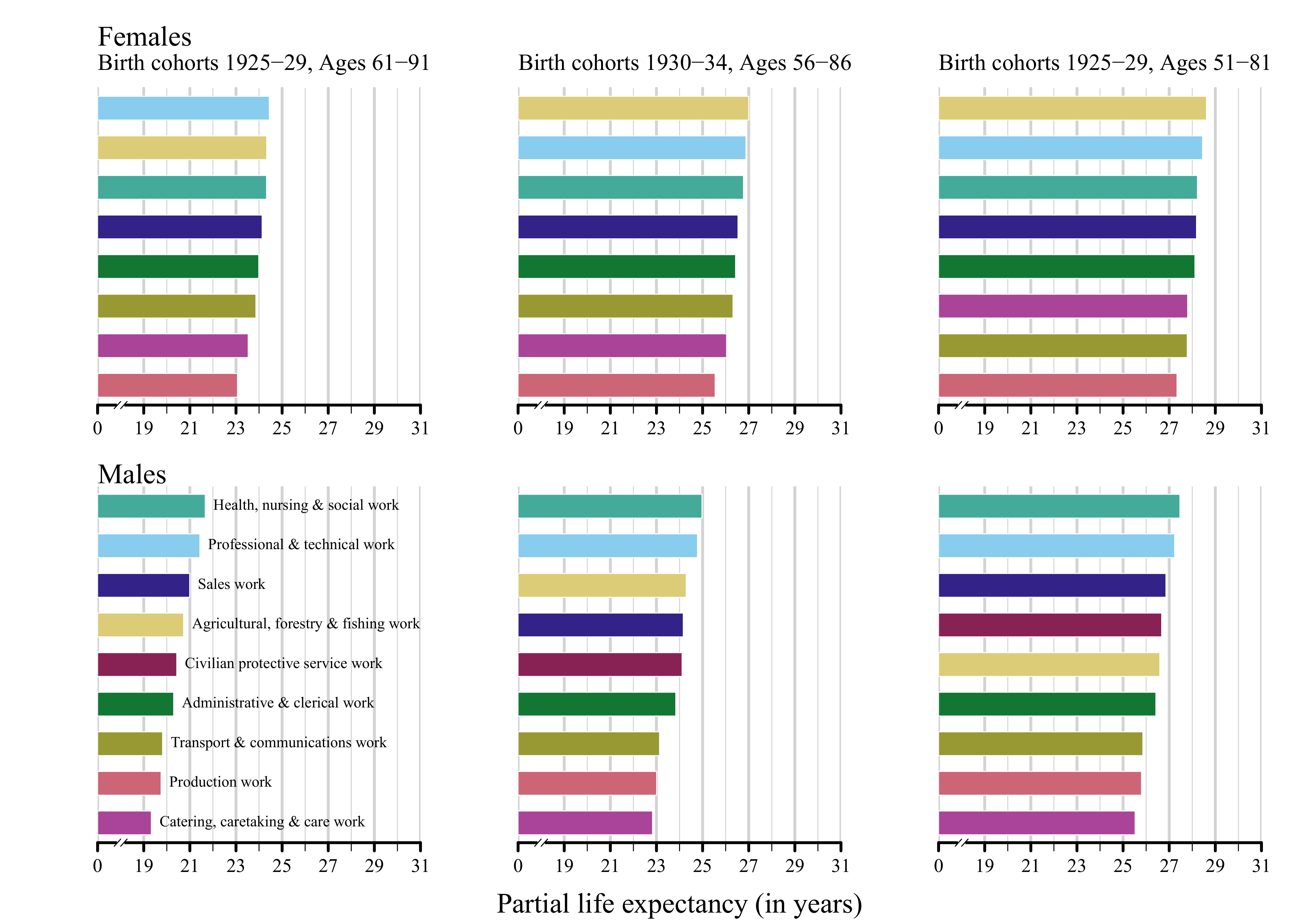

Supplement: sj-jpg-3-sjp-10.1177_14034948221081628 – Supplemental material for Occupational differences in mortality and life expectancy persist after retirement and throughout life [file sj-jpg-3-sjp-10.1177_14034948221081628.jpg]

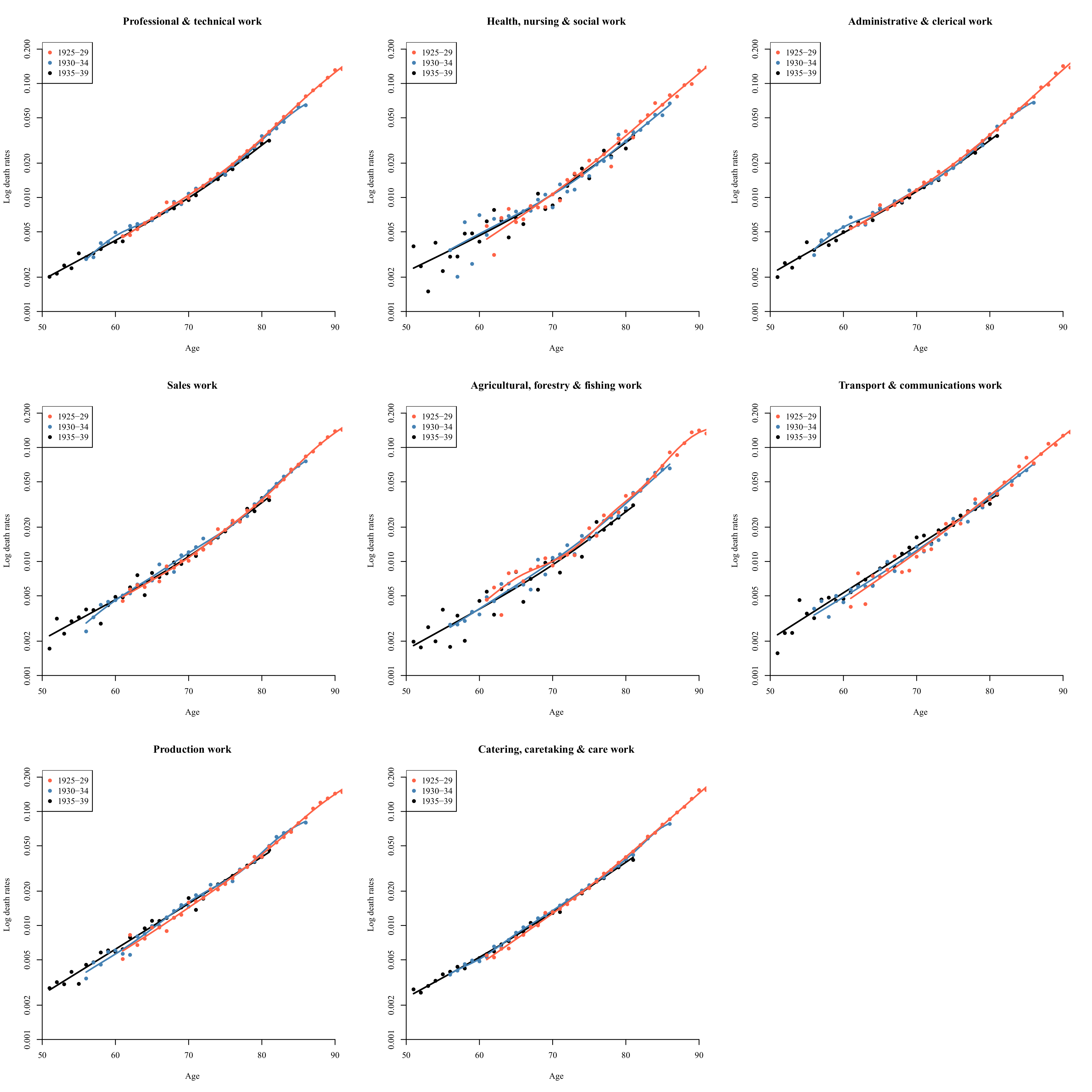

Supplement: sj-jpg-4-sjp-10.1177_14034948221081628 – Supplemental material for Occupational differences in mortality and life expectancy persist after retirement and throughout life [file sj-jpg-4-sjp-10.1177_14034948221081628.jpg]

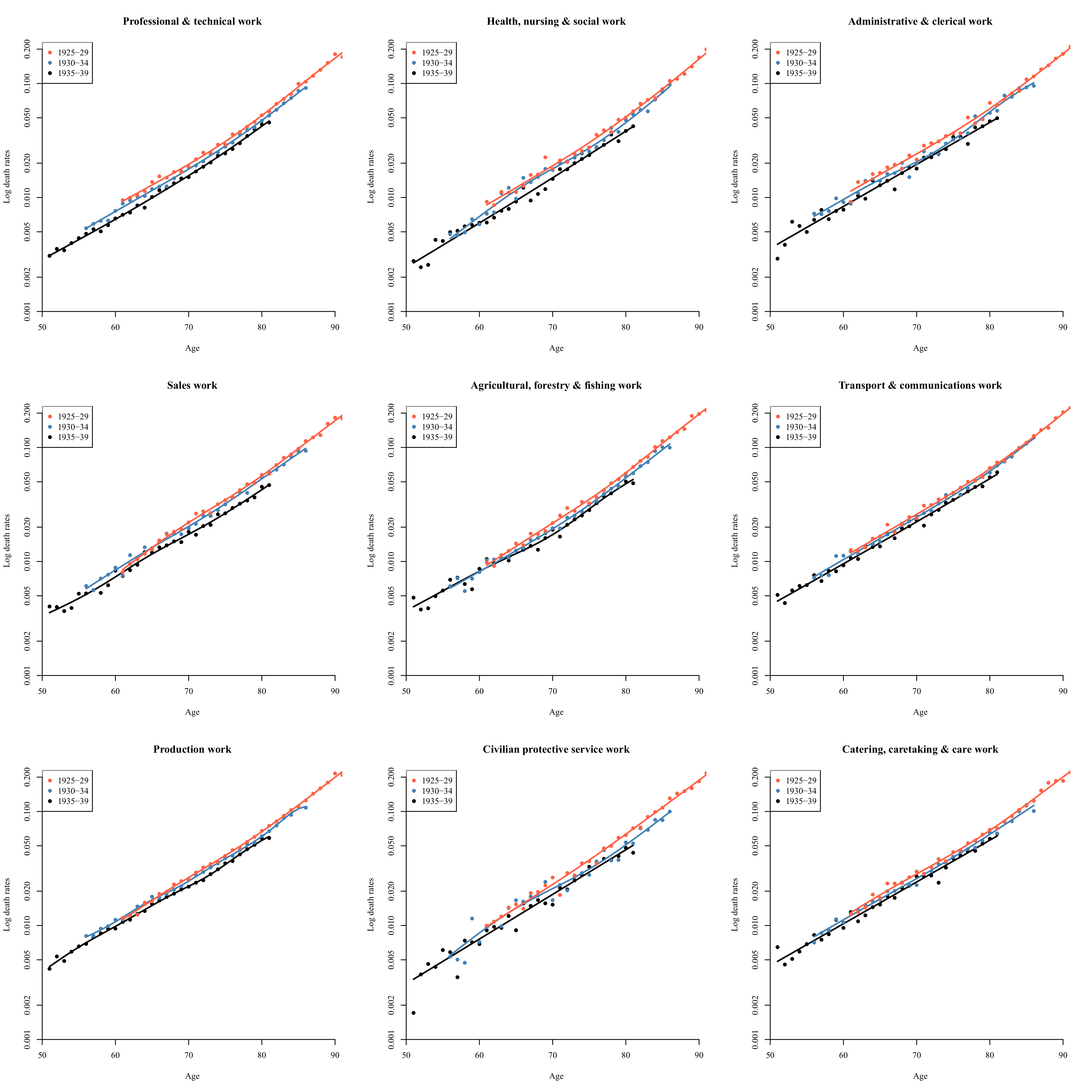

Supplement: sj-jpg-5-sjp-10.1177_14034948221081628 – Supplemental material for Occupational differences in mortality and life expectancy persist after retirement and throughout life [file sj-jpg-5-sjp-10.1177_14034948221081628.jpg]
